# Music models aberrant rule decoding and reward valuation in dementia

**DOI:** 10.1093/scan/nsx140

**Published:** 2017-11-24

**Authors:** Camilla N Clark, Hannah L Golden, Oliver McCallion, Jennifer M Nicholas, Miriam H Cohen, Catherine F Slattery, Ross W Paterson, Phillip D Fletcher, Catherine J Mummery, Jonathan D Rohrer, Sebastian J Crutch, Jason D Warren

**Affiliations:** 1 Dementia Research Centre, UCL Institute of Neurology, University College London, London, UK; 2 Oxford University Clinical Academic Graduate School, University of Oxford, Oxford, UK,; 3 London School of Hygiene and Tropical Medicine, University of London, London, UK

**Keywords:** music, reward, emotion, Alzheimer’s disease, frontotemporal dementia

## Abstract

Aberrant rule- and reward-based processes underpin abnormalities of socio-emotional behaviour in major dementias. However, these processes remain poorly characterized. Here we used music to probe rule decoding and reward valuation in patients with frontotemporal dementia (FTD) syndromes and Alzheimer’s disease (AD) relative to healthy age-matched individuals. We created short melodies that were either harmonically resolved (‘finished’) or unresolved (‘unfinished’); the task was to classify each melody as finished or unfinished (rule processing) and rate its subjective pleasantness (reward valuation). Results were adjusted for elementary pitch and executive processing; neuroanatomical correlates were assessed using voxel-based morphometry. Relative to healthy older controls, patients with behavioural variant FTD showed impairments of both musical rule decoding and reward valuation, while patients with semantic dementia showed impaired reward valuation but intact rule decoding, patients with AD showed impaired rule decoding but intact reward valuation and patients with progressive non-fluent aphasia performed comparably to healthy controls. Grey matter associations with task performance were identified in anterior temporal, medial and lateral orbitofrontal cortices, previously implicated in computing diverse biological and non-biological rules and rewards. The processing of musical rules and reward distils cognitive and neuroanatomical mechanisms relevant to complex socio-emotional dysfunction in major dementias.

## Introduction

Disturbances of complex emotional and social behaviour are a defining hallmark of frontotemporal dementia (FTD) and may be prominent in other neurodegenerative diseases, notably Alzheimer’s disease (AD) (Rascovsky *et al.*, 2011; Sturm *et al.*, 2013; Warren *et al.*, 2013; Sapey-Triomphe *et al.*, 2015; Clark *et al.*, 2016). The spectrum of socio-emotional dysfunction in these diseases is complex and poorly understood; however, certain core themes emerge. These include impaired understanding of social norms and their violation (potentially leading to phenomena such as faux pas, loss of decorum, insensitivity to others’ discomfiture and diffusion of social boundaries: Ibanez and Manes, 2012) and abnormal affective responses to homeostatic signals (leading, e.g. to apathy or abnormal hedonic investment in potentially rewarding, aversive or banal stimuli: Zhou and Seeley, 2014), as well as altered reactivity to emotional cues from complex environments (Sturm *et al.*, 2015) or reduced ability to use semantic regularities in anticipating future events (Bertoux *et al.*, 2014; Irish and Piolino, 2016). These processes might be collectively conceptualized as impaired ‘rule’ decoding and altered reward valuation from socio-emotional and other kinds of signals. Behaviourally, a major unifying goal of such processes is to respond appropriately to salient events in the world at large: ‘salience’ is a property often associated with stimuli that violate expectations (‘rules’) established by prior experience of related stimuli and salient stimuli frequently have reward or aversive potential, according to their intrinsic (biological or cognitive) value and behavioural context (Wallis, 2007; Lang *et al.*, 2009; Dalton *et al.*, 2012; Ibanez and Manes, 2012; Schultz, 2013; Zhou and Seeley, 2014).

Emerging evidence in neurodegenerative populations has underlined the clinical and neuroanatomical relevance of rule and reward processing. Large-scale cerebral networks coding salience (comprising insula, anterior cingulate and orbitofrontal cortex) and reward value (comprising ventral striatal and mesolimbic dopaminergic circuitry) have been implicated in the pathogenesis of socio-emotional phenotypes in FTD (Wallis, 2007; Lang *et al.*, 2009; Pievani *et al.*, 2011; Halabi *et al.*, 2013; Perry *et al.*, 2014, 2015; Zhou and Seeley, 2014; Bocchetta *et al.*, 2016). Altered interactions between salience, reward and temporo-parietal ‘default-mode’ networks underpin socio-emotional dysfunction in AD (Ismail *et al.*, 2008; O’Reilly *et al.*, 2013; Zhou and Seeley, 2014; Fletcher *et al.*, 2015b; Perry *et al.*, 2015; Ahmed *et al.*, 2016). Deficient integration of signal processing with central reward evaluation and autonomic regulatory mechanisms may constitute a common effector pathway in a range of diseases (Zhou and Seeley, 2014; Fletcher *et al.*, 2015a,b). However, the study of rule processing, salience detection and associated reward processing in the dementias poses a number of challenges. These include the complexity of natural socio-emotional scenarios, the diversity of contingent phenomena (spanning the gamut of primary biological and secondary hedonic stimuli) and the difficulty of manipulating relevant stimulus parameters under experimental conditions.

As a candidate model system for analysing rule decoding and reward valuation in dementia, music has a number of attractive attributes. Music is universal, ubiquitous and pleasurable for most listeners; its effects are grounded in cerebral ciricuitry that mediates pattern resolution and hedonic processing, overlapping the distributed networks implicated in FTD and AD (Koelsch *et al.*, 2000; Blood and Zatorre, 2001; Janata *et al.*, 2002; Steinbeis *et al.*, 2006; Pressnitzer *et al.*, 2011; Salimpoor *et al.*, 2011, 2013, 2015; Seger *et al.*, 2013; Clark *et al.*, 2014, 2015a). The socio-emotional resonance of music suggests it may be a fertile model for exploring the building blocks of more complex inter-personal and affective behaviours in daily life (Clark *et al.*, 2015a). Music necessarily unfolds over time, mirroring the dynamic nature of socio-emotional interactions. On the other hand, despite its propensity to engage biological reward circuitry, music is an autonomous signalling system that does not depend on extra-musical associations. Moreover, the characteristics of music that convey salience and hedonic value are highly codified and relatively straightforward to vary experimentally. The ‘rules’ governing harmonic structures are acquired implicitly by normal listeners through exposure to the music of the dominant culture and are engaged rapidly and automatically even during non-attentive listening (Tillmann, 2005; Huron, 2006; Loui *et al.*, 2009; Koelsch *et al.*, 2013; Choi *et al.*, 2014). Indeed, music may be an ideal probe of the interface between cognition and emotion, via the medium of psychological expectancy (Krumhansl, 1997; Huron, 2006).

The relations between rule-based musical expectancy, arousal and valence (perceived pleasantness) are complex and influenced by the specific musical context. The interplay of tension and resolution is a fundamental mechanism underlying a range of psychophysiological (arousal and valence) effects associated with the manipulation of musical structure (Krumhansl, 1997; Steinbeis *et al.*, 2006; Gingras *et al.*, 2016; Tsai and Chen, 2015). Musical pleasure and reward (though separable phenomena) are typically closely correlated (Salimpoor *et al.*, 2013): although ambiguity or surprise in more complex music and musical ‘scenes’ can be intensely pleasurable (Huron, 2006; Pressnitzer *et al.*, 2011), melodies with a harmonic structure that fulfils expectation or resolves ambiguity tend to reduce subjective tension and are usually perceived as subjectively pleasurable or rewarding, while lack of resolution or confounded expectation is associated with increased subjective tension and negative affect (Steinbeis *et al.*, 2006; Gingras *et al.*, 2016; Tsai and Chen, 2015; Tsai *et al.*, 2015). This propensity of music to create psychological expectancy (‘pay-off’ *vs* disappointment) links it naturally to reward valuation, and indeed this is already recognized in the music neuroscience literature (Li *et al.*, 2015; Tsai and Chen, 2015). Reward is a complex, multidimensional psychophysiological construct, but generally entails the potential for consummatory behaviour or active updating of stimulus value, completion or resolution even in the absence of overt goal-directed action (Schultz, 2013; Perry *et al.*, 2015).

From a clinical perspective, altered behavioural responses to music are well documented in many patients with dementia and may stratify FTD and AD syndromes. Recent evidence suggests that behavioural variant (bv)FTD may impair the analysis of musical structure (in particular, anticipation of structure unfolding in time) as well as hedonic valuation of music, whereas semantic dementia (SD) may be associated with relatively preserved processing of musical structure and progressive non-fluent aphasia (PNFA) and AD with relatively preserved hedonic valuation of music (Omar *et al.*, 2010; Weinstein *et al.*, 2011; Hsieh *et al.*, 2012; Downey *et al.*, 2013; Fletcher *et al.*, 2013, 2014, 2015b; Henley *et al.*, 2014; Agustus *et al.*, 2015).

Here we used music as a paradigm to assess rule decoding and reward valuation in the canonical syndromes of FTD and AD, referenced to healthy older individuals. We presented novel melodies that either resolved according to the rules of classical harmony or did not resolve: the behavioural task was to decide whether or not each melody resolved (rule decoding) and to rate its pleasantness (reward valuation). We adopted this tonal expectancy task (rather than simply assessing, e.g. detection of dissonant or scale-deviant notes) because we wished to model the dynamic scenarios of natural, extra-musical, socio-emotional behaviour in daily life: in more complex social scenarios, the potential for reward (here, modelled as harmonic resolution) rather than the delivery of ‘punishment’ (a dissonant note) tends to be the prime mover of behaviour (Milinski and Rockenbach, 2012). At the same time, we wished to avoid any requirement for explicit labelling of musical emotions, a somewhat artificial task that is known to be impaired in FTD syndromes (Omar *et al.*, 2011). We assessed neural correlates of musical rule and reward processing using voxel-based morphometry of patients’ brain MR images. Based on previous evidence, we hypothesized that bvFTD would be associated with abnormalities of both musical rule decoding (categorization of tonally resolved *vs* unresolved melodies) and reward valuation (emotional rating of these melodies) (Omar *et al.*, 2010; Downey *et al.*, 2013; Henley *et al.*, 2014; Agustus *et al.*, 2015; Fletcher *et al.*, 2015b) while SD would show abnormal musical reward valuation despite relatively preserved rule decoding (Omar *et al.*, 2010; Weinstein *et al.*, 2011; Hsieh *et al.*, 2012; Fletcher *et al.*, 2013, 2015b) and PNFA and AD would show abnormal musical rule decoding despite relatively preserved reward valuation (Rohrer *et al.*, 2012; Fletcher *et al.*, 2015b; Golden *et al.*, 2017). Based on previous work in the healthy brain and in patients with focal brain damage, we further hypothesized that harmonic classification of melodies would have a neuroanatomical correlate in superior temporal and prefrontal areas that mediate the analysis of musical and other rule-based patterns (Koelsch *et al.*, 2000, 2013; Janata *et al.*, 2002; Salimpoor *et al.*, 2013, 2015; Seger *et al.*, 2013; Clark *et al.*, 2015a); while the rating of harmonic pleasantness would have a correlate in inferior frontal areas (including orbitofrontal cortex and inferior frontal gyrus) implicated in the affective labelling of music and other stimuli (Blood and Zatorre, 2001; Salimpoor *et al.*, 2011, 2013, 2015; Clark *et al.*, 2014, 2015a) and the integration of semantic and emotional information (Koelsch *et al.*, 2006; Belyk *et al.*, 2017).

## Materials and methods

### Participants

In total 14 patients with typical AD (henceforth, ‘AD’) led by episodic memory decline, 11 patients with bvFTD, 6 patients with SD and 8 patients with PNFA were recruited. All patients fulfilled consensus clinical criteria for the relevant syndromic diagnosis (Gorno-Tempini *et al.*, 2011; Rascovsky *et al.*, 2011; Dubois *et al.*, 2014). Twenty-two healthy older individuals with no history of neurological or psychiatric disorders also participated. Demographic details (including an assessment of past musical training and current listening habits) and clinical characteristics of the study cohort are summarized in Table 1. None of the participants had a history of clinically significant hearing loss or congenital amusia. One patient in the bvFTD group and five patients in the SD group had a history of abnormal craving for music or ‘musicophilia’ (Fletcher *et al.*, 2013). All participants had a comprehensive general neuropsychological assessment (summarized in Table 1) and audiometric screening of peripheral hearing function (adapted from a commercial screening audiometry package; details in Supplementary Material). Brain imaging (MRI/CT) revealed a profile of atrophy compatible with the syndromic diagnosis in all patients; no brain images showed a significant cerebrovascular burden. In total 10 of 11 patients in the AD group for whom CSF was available had a protein marker profile suggesting underlying AD pathology (total CSF tau: beta-amyloid_1-42_ ratio >1, based on local laboratory reference ranges); CSF findings in 11 of 13 patients with other syndromes provided no evidence for underlying AD pathology. At the time of assessment, 13 patients in the AD group were receiving symptomatic treatment with donepezil and 2 with memantine; 1 other patient (in the PNFA group) was receiving donepezil.

**Table 1.** General demographic, clinical and neuropsychological characteristics of participant groups

Characteristic	Healthy controls	bvFTD	SD^a^	PNFA	AD
General					
No. (m:f)	11: 11	9: 2	4: 2	2: 6	8: 6
Age (years)	68.5(5.1)	65.8 (7.6)	66.2 (5.2)	71.5 (7.8)	68.6 (6.7)
Musical training (years)	4.5(3.4)	4.8 (3.4)	4.3 (4.4)	3 (2.5)	4.4 (2.9)
Musical listening (h/wk)	9.7(10.1)	5.5 (4.7)^b^	8.8 (8.6)^b^	5.5 (7.4)	8.6 (11.0)
Education (years)	16.7(2.0)	15.3 (3.4)	14.2 (3.1)	16.9 (2.2)	15 (2.4)
MMSE (/30)	N/A	25 (3.8)	27 (2.5)	23 (9.5)	21 (5.0)
Symptom duration (years)	N/A	9.8 (5.5)	6.3 (1.8)	6.9 (3.7)	6.3 (1.9)
Neuropsychological					
General intellect: IQ					
WASI verbal IQ	119 (7.0)	**91 (16.5)**	**87 (11.5)**	**88 (16.1)**	**98 (14.4)**
WASI performance IQ	121(10.6)	**104 (15.5)**	114 (19.1)	**103 (18.9)**	**90 (21.5)**
NART estimated premorbid IQ	122 (4.7)	**108 (12.2)**	**107 (12.1)**	**104 (15.8)**	**113 (9.0)**
Executive skills					
WASI Block Design (/71)	46 (13.0)	**33 (13.4)**	41 (19.8)	**21 (18.1)**	**18 (13.8)**
WASI Matrices (/32)	25 (4.1)	**21 (5.6)**	24 (6.9)	**20 (6.7)**	**13 (7.8)**
WMS-R digit span forward (/12)	9 (2.1)	9 (2.5)	11 (1.5)	**7 (2.0)**	**6 (2.2)**
WMS-R digit span reverse (/12)	8 (2.0)	7 (2.7)	10 (2.2)	**3 (2.2)**	**5 (1.7)**
D-KEFS Stroop colour (s)	29 (4.2)	**40 (10.3)**	**37 (10.2)**	**67 (20.9)**	**54 (22.5)**
D-KEFS Stroop word (s)	21 (3.7)	**27 (8.0)**	22 (5.3)	**52 (24.6)**	**37 (19.4)**
D-KEFS Stroop interference (s)	58 (17.1)	**81 (36.2)**	62 (27.5)	**149 (37.3)**	**107 (53.2)**
Letter fluency (F: total)^c^	16 (5.5)	**9 (4.6)**	**11 (4.9)**	**4 (2.7)**	**11 (5.0)**
Category fluency (animals: total)	24 (5.4)	**12 (3.8)**	**6 (2.8)**	**10 (3.4)**	**12 (5.4)**
Trails A (s)^d^	32 (9.0)	**45 (17.3)**	35 (20.6)	**69 (37.2)**	**73 (48.0)**
Trails B (s)	73 (20.4)	**148 (81.7)**	89 (48.0)	**233 (67.5)**	**175 (62.9)**
WAIS-R Digit Symbol (total)	56 (10.7)	**34 (8.7)**	**44 (11.2)**	**27 (12.0)**	**20 (15.6)**
Episodic memory					
RMT words (/50)	48 (1.8)	**37 (8.8)**	**33 (6.6)**	47 (3.7)	**30 (5.8)**
RMT faces (/50)	44 (4.1)	**33 (6.1)**	**32 (8.1)**	**37 (5.7)**	**33 (6.4)**
Camden PAL (/24)	20 (2.5)	**9 (6.6)**	**5 (4.5)**	17 (4.5)	**4 (4.0)**
Language skills					
WASI Vocabulary (/80)	71 (3.2)	**50(18.7)**	**42 (17.6)**	**39 (17.9)**	**56 (10.0)**
WASI Similarities (/48)	39 (4.8)	**26(8.4)**	**22 (8.7)**	**28 (7.2)**	**26 (11.4)**
GNT (/30)	26 (2.2)	**8 (9.4)**	**0 (0.8)**	**17 (7.7)**	**16 (6.7)**
NART (/50)	43 (3.6)	**31(11.5)**	**28 (10.5)**	**30 (12.8)**	**36 (7.2)**
Single word repetition (/45)	N/A	N/A	45 (1.0)	33 (15.4)	N/A
Sentence repetition (/10)	N/A	N/A	10 (0.5)	6 (4.4)	N/A
Semantic memory					
BPVS (/150)	148 (1.9)	**128(21.2)**	**120 (14.8)**	**144 (4.8)**	**145 (3.0)**
Synonyms concrete(/25)	N/A	N/A	18 (2.6)	22 (2.9)	N/A
Synonyms abstract(/25)	N/A	N/A	17 (3.2)	21 (3.5)	N/A
Posterior cortical skills					
GDA (/24)	16 (5.0)	13(6.6)	12(8. 4)	**5 (4.1)**	**5 (6.3)**
VOSP Object Decision (/20)	19 (1.6)	**17(1.9)**	17 (2.7)	16 (5.6)	**16 (3.4)**

Notes: Mean (SD) scores are shown unless otherwise indicated; maximum scores are shown after tests (in parentheses). Bold denotes significantly different (*P* < 0.05) to the healthy control group.

AD, patient group with typical Alzheimer’s disease; BPVS, British Picture Vocabulary Scale (Dunn LM et al., 1982); bvFTD, patient group with behavioural variant frontotemporal dementia; D-KEFS, Delis Kaplan Executive System (Delis et al., 2001); GDA, Graded Difficulty Arithmetic (Jackson and Warrington, 1986); GNT, Graded Naming Test (McKenna and Warrington, 1983); MMSE, Mini-Mental State Examination score; N/A, not assessed; NART, National Adult Reading Test (Nelson, 1982); PAL, Paired Associate Learning test (Warrington, 1996); PIQ, performance IQ; PNFA, patient group with progressive non-fluent aphasia; RMT, Recognition Memory Test (Warrington, 1984); SD, patient group with semantic dementia; Synonyms, Concrete and Abstract Word Synonyms Test (Warrington et al., 1998); SD, semantic dementia; VIQ, verbal IQ; VOSP, Visual Object and Spatial Perception Battery (Warrington and James, 1991); WAIS-R, Wechsler Adult Intelligence Scale – Revised (Wechsler, 1981); WASI, Wechsler Abbreviated Scale of Intelligence (Wechsler, 1997); WMS-R, Wechsler Memory Scale - Revised (Wechsler, 1987).

a Three patients (two with musicophilia) had predominantly left-sided temporal lobe atrophy, one (with musicophilia) had predominantly right-sided temporal lobe atrophy and two (also with musicophilia) had more symmetrical, bilateral temporal lobe atrophy.

b includes patients with musicophilia (five in SD group, one in bvFTD group);

c Words generated in 1 min beginning with letter F (Gladsjo et al., 1999).

d Time to complete Trails in seconds (maximum time achievable 2.5 min on task A, 5 min on task B) (Lezak et al., 2004).

The study was approved by the local institutional ethics committee and all participants gave informed consent in accordance with Declaration of Helsinki guidelines.

### Musical assessment

#### Assessment of tonal expectancy

**Fig. 1.**
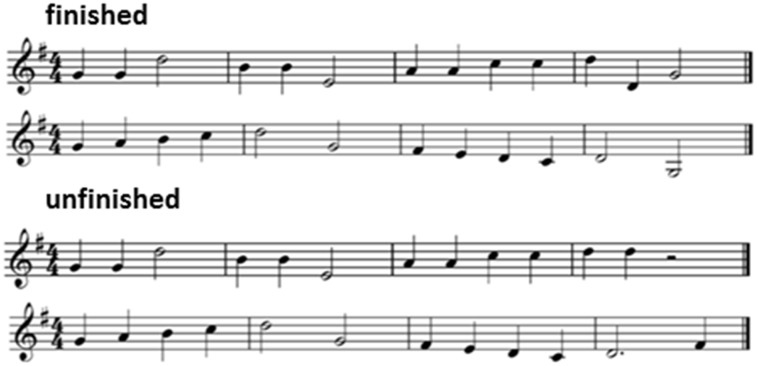
Examples of stimuli used in the music experimental battery: ‘finished’ (tonally resolved) and ‘unfinished’ (tonally unresolved) melodies presented in the tonal (harmonic) expectancy test (see text for further details).

To assess processing of tonal expectancy (musical harmonic) rules and reward value, short monophonic melodies were composed by an experienced musician (O.M.). Melody endings were manipulated such that the melody sounded ‘finished’ (tonally resolved) or ‘unfinished’ (tonally unresolved). Melodies in the ‘finished’ and ‘unfinished’ conditions were closely matched for loudness, length, timbre, instrumentation, key, pitch velocity and tempo. All note sequences were synthesized with piano timbre as digital wavefiles using Logic Pro X. Tempo was fixed at 120 beats/min for all stimuli; melodies varied in length between three and five bars; however, the two conditions did not differ in mean length (number of bars or duration; see Supplementary Material). Examples of stimuli are presented in Figure 1; the complete set is notated in Supplementary Figure S1, together with further details of stimulus preparation and pilot testing.

Melodies were presented in randomized order and the task on each trial was to decide first, if the melody sounded ‘finished’ or ‘unfinished’; and second, to rate how pleasing was the ending of the melody (‘How did the tune leave you feeling?’) on a 5-point Likert scale (1, not at all pleased; 5, very pleased; see Supplementary Figure S2).

#### Assessment of pitch pattern processing

To provide a measure of elementary pitch pattern processing, we adopted a procedure similar to other tests of basic serial pitch discrimination originating in the classic work of Seashore (1919). We assessed participants’ ability to detect pitch direction changes in sequentially presented note pairs; the notes comprising each pair differed in pitch by either a tone or a semitone. Notes had piano timbre and duration 1 s with an inter-note gap of 1 s. Twenty trials (pairs) were presented and the direction of the pitch shift between notes was varied randomly across trials (10 ascending, 10 descending). The task on each trial was to decide if the second note was ‘higher’ or ‘lower’ than the first note.

#### Experimental procedure

Stimuli were presented from a notebook computer running Matlab via headphones (Audio-Technica) at a comfortable listening level (at least 70 dB) in a quiet room. Participants were first familiarised with each test using practice examples to ensure they understood the task and were able to comply reliably. Participant responses were recorded for offline analysis. During the tests no feedback was given about performance and no time limits were imposed. All participants completed the experimental tests comfortably in <30 min.

### Analysis of behavioural data

All behavioural data were analysed using Stata12. Demographic characteristics and neuropsychological data were compared between participant groups using Fisher’s exact test for categorical variables, and for continuous variables, either two sample *t*-tests or Wilcoxon rank-sum tests where *t*-test assumptions were materially violated (e.g. due to skewed data distribution).

Logistic regression was used to analyse accuracy of melody and pitch direction decisions (correct *vs* incorrect) and pleasantness ratings (dichotomized based on a rating ≥3 (‘pleasing’) or <3 (‘not pleasing’) to avoid over-estimating the effect of gradations of pleasantness). For each analysis a global test was first used to jointly compare all groups, followed by pairwise group comparisons where a significant overall group effect was found. An interaction term was included to examine whether there was differential performance between groups for ‘finished’ *vs* ‘unfinished’ melodies. To take account of potentially confounding factors, age, gender, reverse digit span (an index of executive function and specifically, auditory working memory) and (in the assessment of tonal expectancy) performance on the pitch direction task were included as covariates in the regression model.

In separate *post hoc* analyses, we used the non-parametric Spearman coefficient to assess the extent of any correlation between tonal expectancy and pitch direction processing performance and any correlation of musical task performance with years of prior musical training, auditory working memory (reverse digit span) or general executive capacity [Wechsler Abbreviated Scale of Intelligence (WASI) Matrices score, an index of overall disease severity] within the patient cohort.

A threshold *P* < 0.05 was accepted as the criterion for statistical significance in all analyses.

### Brain image acquisition and voxel-based morphometry analysis

Brain MRI data for voxel-based morphometry were acquired for 34 patients (12 AD, 11 bvFTD, 5 SD and 6 PNFA) on a Siemens Trio 3Tesla MRI scanner using a 32-channel phased array head-coil and a sagittal 3D magnetization prepared rapid gradient echo T1-weighted volumetric sequence (echo time/repetition time/inversion time = 2.9/2200/900 ms, dimensions 256 × 256 × 208, voxel size 1.1 × 1.1 × 1.1 mm). Volumetric brain images were assessed visually in all planes to ensure adequate coverage and to exclude artefacts or significant motion. Pre-processing of patient brain MR images was performed using the Segment routine and the DARTEL toolbox of SPM12 (Ashburner, 2007). Further details of imaging analysis are available in Supplementary Material. A study-specific mean brain image template, for displaying results, was created by warping all bias-corrected native space whole-brain images to the final DARTEL template in MNI space and calculating the average of the warped brain.

Using the framework of the general linear model, multiple regression was used to examine associations between regional grey matter volume and accuracy of melody classification as ‘finished’ or ‘unfinished’ (raw score), pleasantness rating of melodies (relative likelihood of rating unfinished melodies as ‘not pleasing’) and pitch direction task performance over the combined patient cohort. In separate design matrices, voxel intensity (an index of grey matter volume) was modelled as a function of each relevant musical behavioural characteristic, including syndromic group membership, age, gender, total intracranial volume and reverse digit span as nuisance covariates in all matrices. For each model, separate contrasts (one-tailed *t*-tests) assessed positive linear associations between grey matter and the parameter of interest across the combined patient cohort. Statistical parametric maps (SPMs) were thresholded at *P* < 0.05 after family-wise error (FWE) correction for multiple voxel-wise comparisons over the whole brain.

## Results

### General participant characteristics

Patient and healthy control groups did not differ in age (*P* = 0.41), gender (*P* = 0.16), educational background (*χ*^2^ = 6.40, *P* = 0.20) or musical training (*χ*^2^ = 1.51, *P* = 0.80) and syndromic groups had similar mean symptom duration (*χ*^2^ = 2.59, *p* = 0.50). Patient groups showed the anticipated profiles of general neuropsychological impairment (Table 1). Peripheral hearing function varied between participant groups [combined audiometric tone detection score, see Supplementary Material; overall *F*(4, 53) = 7.32, *P* < 0.001, Supplementary Table S1); however, the absolute value of the difference was small and there was no significant correlation between peripheral hearing and accuracy on melody classification performance over the entire participant group (rho = −0.09, *P* = 0.50) nor within the combined patient cohort (rho = −0.08, *P* = 0.64).

### Tonal expectancy processing

Group performance profiles on tonal expectancy tasks are summarized in Table 2 and in Supplementary Tables S2 and S3. Individual raw data are presented in Supplementary Figures S3–S5.

**Table 2.** Summary of performance on music cognition tests for patient groups relative to healthy controls

Test characteristic	bvFTD	SD	PNFA	AD
Tonal expectancy task: accuracy classifying melodies				
All	**0.47 (0.25–0.86)**	0.61 (0.30–1.25)	0.66 (0.30–1.44)	**0.55 (0.33–0.91)**
Finished	**0.30 (0.12–0.75)**	0.53 (0.17–1.71)	1.07 (0.43–2.65)	**0.36 (0.18–0.74)**
Unfinished	0.95 (0.45–2.02)	0.79 (0.29–2.14)	**0.32 (0.10–0.99)**	1.16 (0.44–3.01)
Interaction	2.87 (0.91–9.01)^**a**^	0.99 (0.20–4.81)	0.50 (0.18–1.35)	**3.81 (1.17–12.4)^a^**
Tonal expectancy task: rating of melodies as ‘not pleasing’				
All	0.46 (0.14–1.48)	**0.16 (0.04–0.60)^a,^^b^**	1.13 (0.48–2.69)	0.79 (0.36–1.75)
Finished	1.1 (0.34–3.30)	0.52 (0.14–1.92)	0.84 (0.31–2.26)	1.07 (0.42–2.75)
Unfinished	0.32 (0.09–1.10)	**0.10 (0.03–0.36)**	1.45 (0.42–4.97)	0.72 (0.28–1.72)
Interaction	**0.30 (0.11–0.85)^a^**	**0.19 (0.09–0.39)^a,^^b^**	1.72 (0.39–7.61)	0.65 (0.25–1.71)
Pitch direction task				
Accuracy	**0.40 (0.18–0.91)**	**0.34 (0.13–0.87)**	0.51 (0.12–2.10)	0.57 (0.12–2.69)

Notes: For tonal expectancy test data, odds ratios (95% confidence intervals) are shown for correctly classifying melodies as ‘finished’ *vs* ‘unfinished’ and for rating the endings of melodies as ‘not pleasing’ *vs* ‘pleasing’ (see text), relative to the healthy control group; ‘interaction’ here represents the odds of a score difference for ‘finished’ *vs* ‘unfinished’ melodies, expressed for each patient group relative to healthy controls. For the pitch direction task, the OR represents the relative accuracy of pitch direction labelling relative to healthy control performance. For all odds ratios, confidence intervals including 1 indicate no significant difference between that patient group and healthy controls for the parameter of interest. For all comparisons, patient group performance profiles that differed significantly (*P* < 0.05) from the healthy control group are shown in bold; ^**a**^significantly different (*P* < 0.05) from PNFA group; ^**b**^significantly different (*P* < 0.05) from AD group; AD, patient group with typical Alzheimer’s disease, bvFTD, patient group with behavioural variant frontotemporal dementia; PNFA, patient group with progressive non-fluent aphasia; SD, patient group with semantic dementia.

There was evidence of an overall group performance difference in the odds of correctly classifying melodies as ‘finished’ or ‘unfinished’ (*P* = 0.003; Table 2 and Supplementary Figure S3). Relative to the healthy control group, the bvFTD and AD groups each showed overall significantly less accurate classification of melodies (*P* < 0.05), driven principally by incorrect classification of ‘finished’ melodies (Table 2). The SD and PNFA groups did not show a significant deficit of melody classification. There were no significant differences between syndromic groups. Whereas healthy controls were equivalently accurate in classifying ‘finished’ and ‘unfinished’ melodies [odds ratio (OR) = 1.4 (95% confidence interval (CI) 0.7–2.9), *P* = 0.38], the AD group was significantly less accurate classifying ‘finished’ than ‘unfinished’ melodies (*P* < 0.001). Across the patient cohort, accuracy of melody classification did not correlate with prior musical training (rho = 0.19, *P* = 0.25) or auditory working memory (reverse digit span, rho = 0.20, p = 0.23) but was significantly correlated with a general measure of non-verbal executive function (WASI Matrices score, rho = 0.46, *P* = 0.003).

There was further evidence of an overall group difference in pleasantness ratings of ‘unfinished’ *vs* ‘finished’ melodies (*P* < 0.0001; Table 2 and Supplementary Figure S4). The healthy control group was significantly more likely to rate ‘unfinished’ than ‘finished’ melodies as ‘not pleasing’ [OR = 7.7; CI 3.8–15.7]; the ranges of individual raw healthy control ratings for ‘finished’ and ‘unfinished’ melodies overlapped (Supplementary Figure S4 and Supplementary Table S2), suggesting that controls did not simply rate melodies to align explicitly with their melody classification. Patient groups showed a qualitatively similar profile, in that all groups tended to rate ‘finished’ melodies as more pleasant than ‘unfinished’ melodies (Supplementary Figure S4). However, both the bvFTD and SD groups showed significantly less discrepant pleasantness rating profiles than did the healthy control group (*P* < 0.05) and the SD group was also significantly less likely than healthy controls to rate ‘unfinished’ melodies as ‘not pleasing’ (*P* < 0.001), while the PNFA and AD groups showed a profile comparable to healthy controls. Comparing disease groups revealed significant differences between the pleasantness rating profiles of the bvFTD and SD groups *vs* the PNFA and AD groups (*P* < 0.05). Patient group pleasantness rating profiles were similar for the complete melody set and when restricted to those melodies correctly classified as ‘finished’ or ‘unfinished’ (compare Supplementary Figures S4 and S5, see Supplementary Table S3): both the bvFTD and SD groups were significantly less likely than healthy controls to rate correctly classified melodies as ‘not pleasing’ (*P* < 0.05) while the PNFA and AD groups rated correctly classified melodies similarly to healthy controls.

### Pitch direction processing

Relative to healthy controls, a deficit of pitch direction processing was evident in the bvFTD group (*P* = 0.03) and SD group (*P* = 0.03), but not the PNFA group (*P* = 0.35) or AD group (*P* = 0.48) (Table 2 and Supplementary Figure S3). Within the patient cohort, performance on the pitch direction and tonal expectancy tasks showed a borderline significant correlation (*P* = 0.05, rho = 0.31; analyses of tonal expectancy performance were adjusted for pitch direction score). Performance on the pitch direction task was significantly correlated with prior musical training (rho = 0.58, *P* = 0.0002) but not with executive measures (reverse digit span, rho = 0.26, *P* = 0.11; WASI Matrices score, rho = 0.28, *P* = 0.08).

### Neuroanatomical associations

**Fig. 2.**
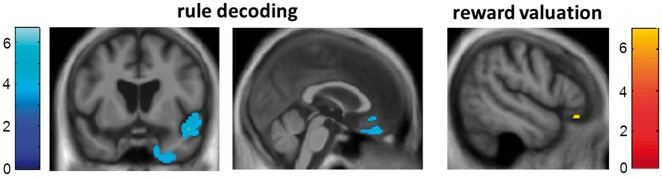
Statistical parametric maps (SPMs) of regional grey matter volume positively associated with tonal expectancy parameters in the combined patient cohort are presented (see also Table 3); t scores are coded on the colour bars. Grey matter associations of accuracy of melody classification signify musical rule decoding, indicated in blue; grey matter associations of melody pleasantness rating signify musical reward valuation, indicated in red-orange (see text for details). SPMs are overlaid on coronal (left) and sagittal (middle, right) sections of the normalized study-specific T1-weighted mean brain MR image, selected to highlight right anterior superior temporal and entorhinal cortex (left), right medial orbitofrontal cortex (middle) and left inferior frontal gyrus pars orbitalis (right). SPMs are thresholded for display purposes at *P* < 0.001 uncorrected, however local maxima of areas shown were each significant at *P* < 0.05 after FWE correction for multiple voxel-wise comparisons over the whole brain (see Table 3).

Significant neuroanatomical associations are summarized in Table 3 and SPMs are presented in Figure 2.

**Table 3.** Summary of grey matter associations of tonal expectancy processing in patient cohort

Region	Peak coordinate (mm)			Z score	*P* value
	x	y	z		
Accuracy of classifying melodies					
Entorhinal cortex	24	0	–50	5.22	0.008
Anterior superior temporal gyrus	48	3	–18	4.94	0.025
Anterior superior temporal sulcus	56	–10	–8	4.91	0.029
Medial orbitofrontal cortex	4	40	−22	4.92	0.028
Pleasantness rating of melodies					
Inferior frontal gyrus (pars orbitalis)	−51	33	−15	5.69	0.001

Notes: The Table shows statistically significant positive associations between grey matter volume and accuracy of classifying melodies (‘finished’ *vs* ‘unfinished’) and pleasantness rating of melodies (likelihood of rating unfinished melodies as ‘not pleasing’), based on a voxel-based morphometric analysis of brain MR images for the combined patient cohort. Local maxima coordinates are presented with coordinates in MNI standard stereotactic space, thresholded at *P* < 0.05 after FWE correction for multiple voxel-wise comparisons over the whole brain. No significant associations were identified for the pitch direction task at the prescribed threshold.

Significant associations between grey matter atrophy and impaired melody classification accuracy over the combined patient cohort were identified in right entorhinal cortex, anterior superior temporal gyrus and sulcus and medial orbitofrontal cortex, thresholded at *P* < 0.05_FWE_ after correction for multiple voxel-wise comparisons over the whole brain. A significant association between grey matter atrophy and abnormal pleasantness rating of melodies (a tendency to rate unresolved melodies as less unpleasant than healthy controls) was identified in left inferior frontal gyrus (pars orbitalis), thresholded at *P* < 0.05_FWE_ after correction for multiple voxel-wise comparisons over the whole brain. No significant grey matter associations of pitch direction processing were identified for the combined patient cohort at the prescribed threshold.

## Discussion

Here we have shown that music models impairments of rule and reward processing in cardinal syndromes of FTD and AD. When compared with healthy controls, patients with bvFTD had both less accurate classification of melodies based on harmonic structure (impaired rule decoding) and altered affective responses to harmonic completion (abnormal reward valuation); patients with SD had abnormal reward valuation despite preserved rule decoding; patients with AD had normal reward valuation despite impaired rule decoding; and patients with PNFA showed a relatively normal profile. Taken together, these profiles argue for dissociable signatures of musical rule decoding and reward valuation across dementia syndromes.

Melody classification in the bvFTD and AD groups was impaired after taking elementary pitch pattern perception (performance on the pitch direction task) into account and classification accuracy did not correlate with auditory working memory or prior musical training. It is therefore likely that the tonal expectancy test indexed a relatively specific impairment of musical rule processing. Such cognitive modularity may have contributed to the (on face value, somewhat surprising) preserved performance of the present PNFA group on melody classification, despite other evidence for impaired syntactical and pitch pattern deficits in this syndrome (Rohrer *et al.*, 2012; Golden *et al.*, 2017). On the other hand, tonal expectancy was aligned with general executive capacity, suggesting that this musical task may track disease severity. Abnormalities of musical reward valuation in bvFTD and SD were evident even in the context of correct rule decoding (for correctly classified melodies), further demonstrating that the processes of rule decoding and reward valuation are dissociable. The profiles of musical valuation exhibited by these syndromic groups were qualitatively similar to healthy controls despite quantitative differences, raising the possibility that more complex effects (such as biased ‘gain’ or altered precision of reward expectancy) might be revealed by larger cohort studies with scope to further refine the model of musical hedonic valuation in these diseases.

The musical profiles identified here resonate with behaviours exhibited by patients with FTD and AD in various other experimental and social contexts. Impaired rule-based processing on semantic categorization tasks has been demonstrated in bvFTD and AD (Grossman *et al.*, 2001, 2003). More pertinently, these patients show impaired anticipation of future events based on previously experienced regularities (Irish *et al.*, 2012; Irish and Piolino, 2016): a general mechanism for impaired decision-making. Patients with bvFTD show markedly impaired detection of rule violations in social scenarios (faux pas) and increased risk taking behaviour and reduced anticipation of future regret (aberrant reward prediction) (Rahman *et al.*, 1999; Torralva *et al.*, 2007; Bertoux *et al.*, 2014; Bora *et al.*, 2015). The present findings support previous evidence that detection of salience (rule violation) in socio-emotional contexts is generally impaired in bvFTD while perceptual and cognitive detection of salient events may be disengaged from affective reactivity in this syndrome (Sturm *et al.*, 2006; Chiong *et al.*, 2013; Clark *et al.*, 2015b, 2016). In contrast, patients with AD have difficulty using rules to make decisions about future outcomes, but remain sensitive to affective outcomes (reward value) (Delazer *et al.*, 2007, Dohnel *et al.*, 2008; Sinz *et al.*, 2008). AD and PNFA are substantially less likely than patients with bvFTD to exhibit strikingly aberrant responses to social rule violations (Bora *et al.*, 2015; Clark *et al.*, 2016), perhaps reflecting the relative extent to which affective awareness is preserved in these syndromes. In SD, decision-making based on modelling of future events depends critically on semantic function (Irish *et al.*, 2011, 2012; Irish and Piolino, 2016): music (in contrast to most other rule-based systems) does not rely on extraneous semantic associations, perhaps accounting for the preserved ability to use musical rules exhibited by patients with SD here and in previous studies (Hailstone *et al.*, 2009; Omar *et al.*, 2010; Weinstein *et al.*, 2011). Nevertheless (in line with the present profile), SD is frequently associated with abnormal valuation of biological and other rewards, including music (Fletcher *et al.*, 2013, 2015a,b; Ahmed *et al.*, 2014, 2015, 2016).

The neuroanatomical profiles identified here underline the involvement of brain networks mediating rule analysis, rule violation (salience) detection and reward evaluation in these diseases (Perry *et al.*, 2014, 2015). Impaired tonal expectancy (classification of melodies) was associated with grey matter loss in right anterior superior and inferior temporal cortices and medial orbitofrontal cortex. In line with previous work (Peretz *et al.*, 2001; Koelsch *et al.*, 2006, 2013; Khalfa *et al.*, 2008; Fujisawa and Cook, 2011; Hailstone *et al.*, 2011; Omar *et al.*, 2011; Hsieh *et al.*, 2012; Salimpoor *et al.*, 2013; Seger *et al.*, 2013; Bonfiglio *et al.*, 2015), this network may link cortical mechanisms mediating the structural analysis of melodies and harmonic hierarchies with paralimbic and orbitofrontal mechanisms mediating the cognitive representation and anticipation of musical emotion and reward. More generically, antero-medial temporal areas store previously learned knowledge and templates about sensory objects and regularities whereas prefrontal cortices implement and assess violations in rule-based algorithms (Michelon *et al.*, 2003; Christensen *et al.*, 2011; Nazimek *et al.*, 2013; Cohen, 2014; Gauvin *et al.*, 2016; Henderson *et al.*, 2016). Altered valuation of musical reward (affective rating of melodies) was associated with grey matter loss in left inferior frontal gyrus pars orbitalis. Functionally, this region behaves as a subdivision of lateral orbitofrontal cortex and links cortical mechanisms analysing hierarchical and rule-based patterns [such as linguistic and musical ‘syntax’) with mechanisms representing reward value (Belyk *et al.*, 2017); it has been implicated previously in representing musical tension associated with violation of harmonic expectancy and anticipation of musical rhythms (Lehne *et al.*, 2014; Vuust *et al.*, 2014)]. We did not find neuroanatomical correlates in striatal or other subcortical structures previously implicated in processing musical reward (Omar *et al.*, 2011; Salimpoor *et al.*, 2013; Li *et al.*, 2015). Previous studies have generally employed music familiar to the participants or explicit (e.g. monetary) valuation of music, perhaps implying that other motivational, emotional or subjective factors engage these subcortical mechanisms.

Impairments of social and emotional functioning remain difficult to assess in cognitively impaired patients, limiting the identification of relevant biomarkers and the design of effective interventions. Taken together, the present findings suggest that music may provide a useful paradigm for deconstructing complex behavioural disturbances in FTD and AD. Beyond its universality and relative tractability, music (as a dynamic stimulus that unfolds in time) inherently entails predictive coding: the anticipation of musical structure based on internalized models that are continually updated (Huron, 2006). Aberrant predictive coding has emerged as a key organizing principle in computational accounts of major psychiatric disorders (Friston *et al.*, 2014; Adams *et al.*, 2016) and may play a similarly fundamental role in the phenomenology of neurodegenerative diseases characterized by deficient simulation of future events and consequences (Rahman *et al.*, 1999; Torralva *et al.*, 2007; Irish *et al.*, 2012; Bertoux *et al.*, 2014; Irish and Piolino, 2016). Music might generate novel dynamic biomarkers that can detect and track alterations in this core disease mechanism in the dementias, analogous to the next generation disease markers recently proposed for psychiatric illness (Friston *et al.*, 2014; Adams *et al.*, 2016).

This study suggests a number of directions for future work. Larger patient cohorts will be required to characterize the specificity of musical reward phenotypes for particular diseases while taking account of intrinsic individual variation in the cognitive decoding and hedonic valuation of music (Clark *et al.*, 2014; Salimpoor *et al.*, 2015; Sachs *et al.*, 2016; Supplementary Figures S3–S5). Voxel-based morphometry is necessarily an associational technique and in clinical populations the associations observed are ‘windowed’ by the distribution of tissue damage imposed by the target disease. A complete picture of neurodegenerative musical phenotypes will entail functional neuroimaging and connectivity approaches that can delineate network-level effects directly (Salimpoor *et al.*, 2013; Sachs *et al.*, 2016), in addition to autonomic and other physiological metrics that may reveal disease-linked dissociations between implicit and explicit coding of musical reward (Balconi *et al.*, 2015; Fletcher *et al.*, 2015c; Sturm *et al.*, 2015). The potential relevance of music as a biomarker will only be defined by longitudinal studies, including pre-symptomatic genetic cohorts. Assessment of hearing functions more generally (including the role of peripheral hearing) also warrants further study in common dementias; although peripheral hearing changes did not materially affect the key findings here, more elementary auditory processes may nevertheless be relevant to the broader characterization of these syndromes and as biomarkers in their own right (Hardy *et al.*, 2016). Acknowledging these caveats, the present findings provide a case for music as a useful probe of aberrant rule and reward mechanisms in the dementias. We propose a reappraisal of music as a unique window on complex behaviours in neurodegenerative disease.

## Supplementary data

Supplementary data are available at *SCAN* online.

## Supplementary Material

Supplementary Figures and Tables
