# Supplementary material for: Music models aberrant rule decoding and reward valuation in dementia
Source: Soc Cogn Affect Neurosci. 2017 Nov 24;13(2):192–202. doi: 10.1093/scan/nsx140 (PMC5827340; doi:10.1093/scan/nsx140)
Supplement: Supplementary Figures and Tables [file nsx140_supp.docx]

**Supplementary material: Music models aberrant rule decoding and reward valuation in dementia, by CN Clark et al**

## Peripheral hearing assessment and analysis

## Peripheral hearing ability was assessed in each participant using pure tone audiometry, administered via headphones from a notebook computer in a quiet room. The procedure was adapted from a commercial screening audiometry software package (AUDIO- CDTM®, http://www.digital-recordings.com/audiocd/audio.html). Five frequency levels (500, 1000, 2000, 3000, 4000 Hz) were assessed: at each frequency, participants were presented with a continuous tone that slowly and linearly increased in intensity. Participants were instructed to indicate as soon as they were sure they could detect the tone; this response time was measured and stored for offline analysis. Hearing was assessed in the right ear.

Tone detection thresholds on audiometry screening were analysed using a multiple linear regression model. Robust standard errors were used as the assumption of homogeneity of variance was violated. The main effect of patient group was assessed whilst controlling for age. A combined log transformed (owing to a skewed non-normal distribution) audiometry score using the sum of detection thresholds for all frequencies was derived as an overall measure of peripheral hearing function to test for associations with performance on the experimental tests of music processing.

**Tonal expectancy test**

Melodies (see Figure S1) were composed in accord with the rules of Western classical harmony, based on motifs that commenced on either the tonic or dominant degree of the scale to establish the tonal centre and of sufficient length to establish stylistically congruous harmonic progressions. ‘Finished’ melodies expressed perfect cadences (dominant – tonic) in the final bar whilst ‘unfinished’ melodies implied either imperfect cadences (ending on the dominant), interrupted cadences (dominant – submediant), or incomplete perfect cadences (dominant-leading note). Based on data in a pilot group of 15 healthy older individuals who were separate from the healthy controls used in the main experimental cohort (mean age 61 years, range 51 to 74 years; 9 female), a subset of 24 melodies (12 pairs) from an initial set of 40 were selected to comprise the final stimulus set. The criterion for inclusion of a melody in the final set was >75% consensus agreement across the pilot control cohort as to whether that melody sounded ‘finished’ or ‘unfinished’. The final stimulus set covered a range of keys (19 major, 5 minor) and time signatures (17 in 4/4, four in 3/4, three in 5/4). Average loudness (root-mean-square intensity) of the sound files was fixed in Matlab®.

### Brain imaging analysis

### Normalisation, segmentation and modulation of grey and white matter images used default parameter settings, with a smoothing Gaussian kernel of full-width-at-half-maximum 6mm. Smoothed segments were warped into MNI space using the “Normalise to MNI” routine. In order to adjust for individual differences in global grey matter volume during subsequent analysis, total intracranial volume was calculated for each participant by summing grey matter, white matter and cerebrospinal fluid volumes following segmentation of all three tissue classes.

To help protect against voxel drop-out due to marked local regional atrophy, a customised explicit brain mask was made based on a specified ‘consensus’ voxel threshold intensity criterion (Ridgway GR et al. Neuroimage 2009), whereby a particular voxel was included in the analysis if grey matter intensity at that voxel was >0.1 in >70% of participants (rather than in all participants, as with the default SPM mask). The mask was applied to the smoothed grey matter segments prior to statistical analysis

**Table S1.** Summary of peripheral hearing function in participant groups

| **Audiometry parameter** | **Healthy controls** | **bvFTD** | **SD** | **PNFA** | **AD** |
| --- | --- | --- | --- | --- | --- |
| Summed score | 10.9 (0.5) | 11.5 (0.4) | 11.1 (0.2) | 11.3 (0.6) | 10.8 (0.7) |
| Comparison with healthy controls: p value |  | **<0.001** | 0.08 | 0.10 | 0.68 |

Overall mean (standard deviation) natural log summed audiometry scores (based on detection times (msec) over tested frequencies) for each participant group and pairwise regression comparisons (covaried for age) of patient groups versus the healthy control group are presented. Significant (p<0.05) patient group differences relative to healthy controls are indicated in bold. AD, patient group with Alzheimer’s disease; bvFTD, patient group with behavioural variant frontotemporal dementia; PNFA, patient group with progressive nonfluent aphasia. SD, patient group with semantic dementia.

**Table S2.** Summary of performance on music cognition tests for all participant groups: raw data

| **Test characteristic** | **Healthy controls** | **bvFTD** | **SD** | **PNFA** | **AD** |
| --- | --- | --- | --- | --- | --- |
| ***Tonal expectancy task: accuracy classifying melodies*** | | | | | |
| Accuracy classifying melodies | | | | | |
| All (/24) | 0.89 (0.31) | **0.75 (0.43)** | 0.84 (0.37) | 0.81 (0.39) | **0.78 (0.42)** |
| Finished (/12) | 0.88 (0.33) | **0.64 (0.48)** | 0.82 (0.39) | 0.84 (0.37) | **0.65 (0.48)** |
| Unfinished (/12) | 0.91 (0.29) | 0.87 (0.34) | 0.86 (0.35) | **0.78 (0.42)** | 0.90 (0.29) |
| ***Tonal expectancy task: pleasantness rating of melodies*** | | | | | |
| All | 3.2 (1.2) | 3.4 (1.0) | **3.8 (1.0)** | 3.0 (1.3) | 3.2 (1.3) |
| Finished | 3.9 (1.1) | 3.7 (1.0) | 4.1 (0.9) | 3.7 (1.1) | 3.7 (1.2) |
| Unfinished | 2.5 (1.0) | 3.2 (0.9) | **3.5 (1.0)** | 2.1 (1.0) | 2.7 (1.2) |
| ***Pitch direction task*** | | | | | |
| Accuracy (/20) | 0.95 (0.23) | **0.84 (0.37)** | **0.87 (0.34)** | **0.86 (0.35)** | **0.87 (0.34)** |

Accuracy scores are shown as mean (standard deviation) proportion correct (where 0.5 is chance performance; maximum raw scores are indicated in parentheses for each condition); pleasantness ratings are mean (standard deviation) Likert scores on scale 1 (not at all pleasing) to 5 (very pleasing). Note that pitch direction accuracy scores here differ slightly from the odds ratios reported in Table 2, as the latter are adjusted for covariates. Bold denotes a value significantly different (p<0.05) to healthy control group; AD, patient group with typical Alzheimer’s disease; bvFTD, patient group with behavioural variant frontotemporal dementia; PNFA, patient group with progressive nonfluent aphasia; SD, patient group with semantic dementia. See text for further details of conditions and Figures S3 and S4 for individual data plots.

**Table S3**. Summary of melody pleasantness ratings for patient groups relative to healthy controls: correctly classified melodies only

| **Test characteristic** | **bvFTD** | **SD** | **PNFA** | **AD** |
| --- | --- | --- | --- | --- |
| No. of stimuli analysed  (healthy control n=472) | 199 | 121 | 156 | 261 |
| Odds ratios: |  |  |  |  |
| All melodies | **0.28 (0.08-0.98)^a^** | **0.12 (0.02-0.57)^a^** | 1.93 (0.82-4.54) | 0.79 (0.27-1.50)**^a^** |
| Finished | 0.17 (0.03-1.07) | 0.69 (0.09-5.29) | 0.80 (0.19-3.45) | 0.37 (0.04-3.44) |
| Unfinished | 0.29 (0.08-1.08)**^a^** | **0.08 (0.02-0.32) ^a,b^** | 3.08 (0.85-11.13) | 0.70 (0.27-1.81)**^a^** |
| Interaction | 1.77 (0.37-8.55) | **0.12 (0.02-0.57)*** | 3.84 (0.48-30.90) | 1.90 (0.18-19.95) |

This table presents a subanalysis of pleasantness ratings restricted to those melodies that were correctly classified by each participant; the total number of correct stimuli analysed for each group is indicated. Odds ratios (95% confidence intervals) are shown for rating the endings of melodies as ‘not pleasing’ versus ‘pleasing’ (see text), relative to the healthy control group; ‘interaction’ here represents the odds of a score difference for ‘finished’ versus ‘unfinished’ melodies, expressed for each patient group relative to healthy controls. Confidence intervals including 1 indicate no significant difference between that patient group and healthy controls. For all comparisons, patient group profiles that differed significantly (p<0.05) from the healthy control group are shown in bold; a, significantly different (p<0.05) from PNFA group; b, significantly different (p<0.05) from AD group; *significantly different (p<0.05) from all other disease groups; AD, patient group with typical Alzheimer’s disease, bvFTD, patient group with behavioural variant frontotemporal dementia; PNFA, patient group with progressive nonfluent aphasia; SD, patient group with semantic dementia.

**Figure S1.** Stimuli used to assess tonal expectancy

**
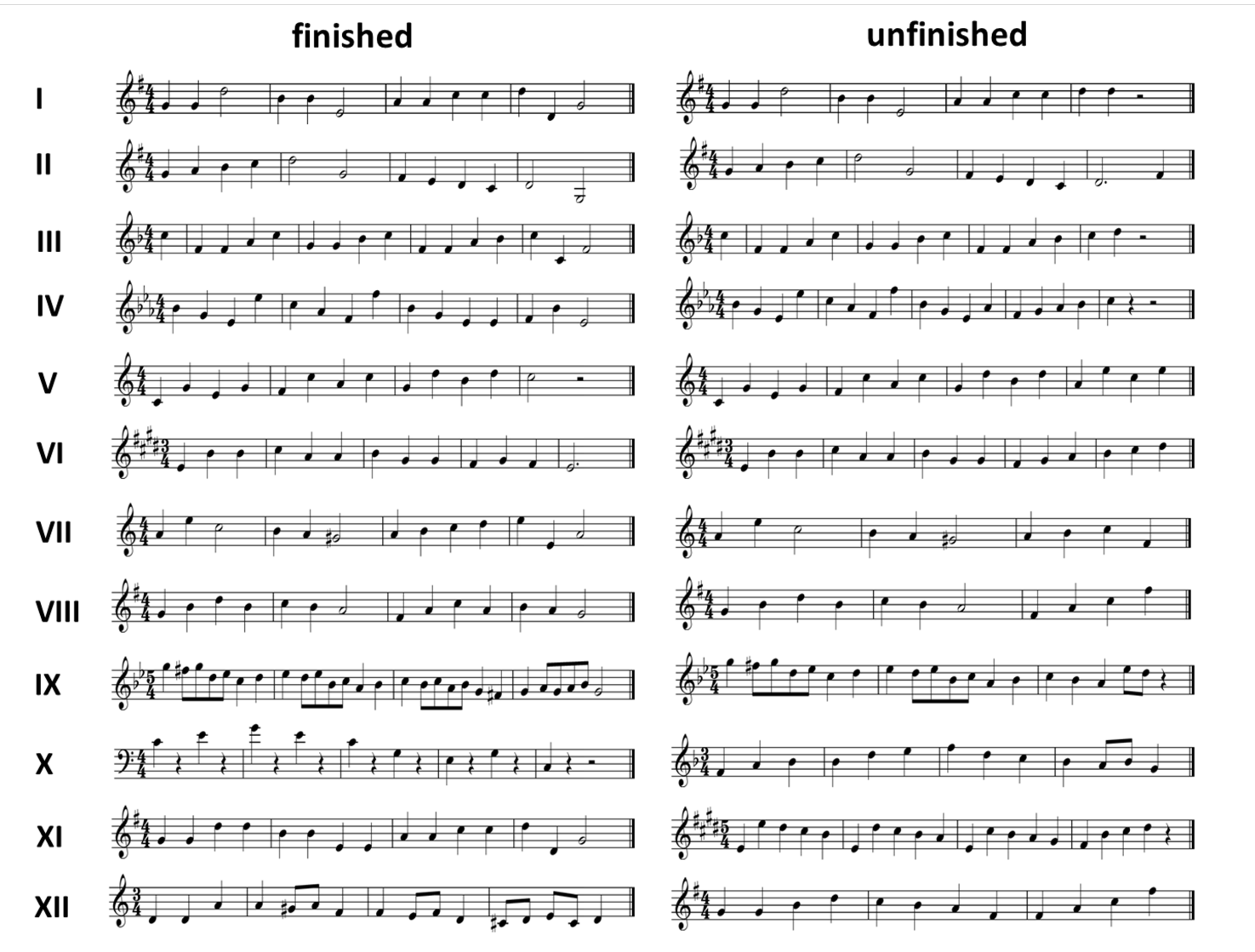
**

Stimuli were administered as digital wavefiles with piano timbre, in randomised order. Melodies in rows I to IX were created by varying a harmonic motif common to each ‘finished’ (tonally resolved) – ‘unfinished’ (tonally unresolved) pair; stimuli in rows X to XII were created from unique harmonic motifs. ‘Finished’ and ‘unfinished’ melodies when compared using two-tailed t tests did not differ in number of bars (‘finished’, mean 4.1 (standard deviation 0.29); ‘unfinished’, mean 3.8 (standard deviation 0.72); p=0.27) or overall duration (‘finished’, mean 0.09 (standard deviation 0.01) seconds; ‘unfinished’, mean 0.08 (standard deviation 0.01) seconds; p=0.5). See text for further details

**Figure S2.** Visual scale used by participants to rate pleasantness of musical stimuli

**
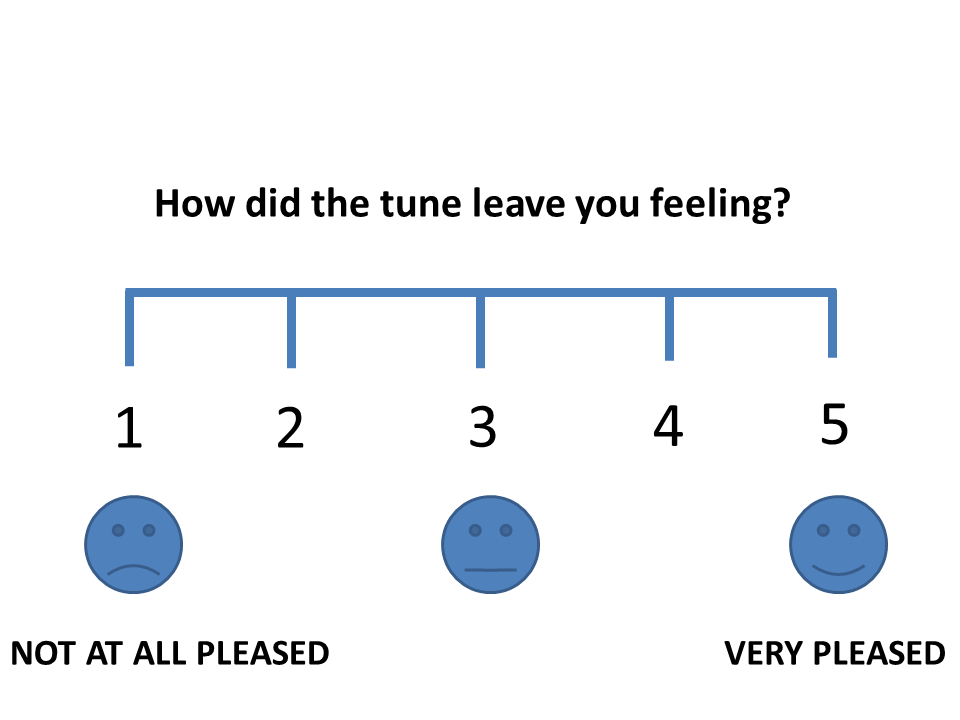
**

**Figure S3**. Individual accuracy scores for tonal expectancy and pitch direction tasks


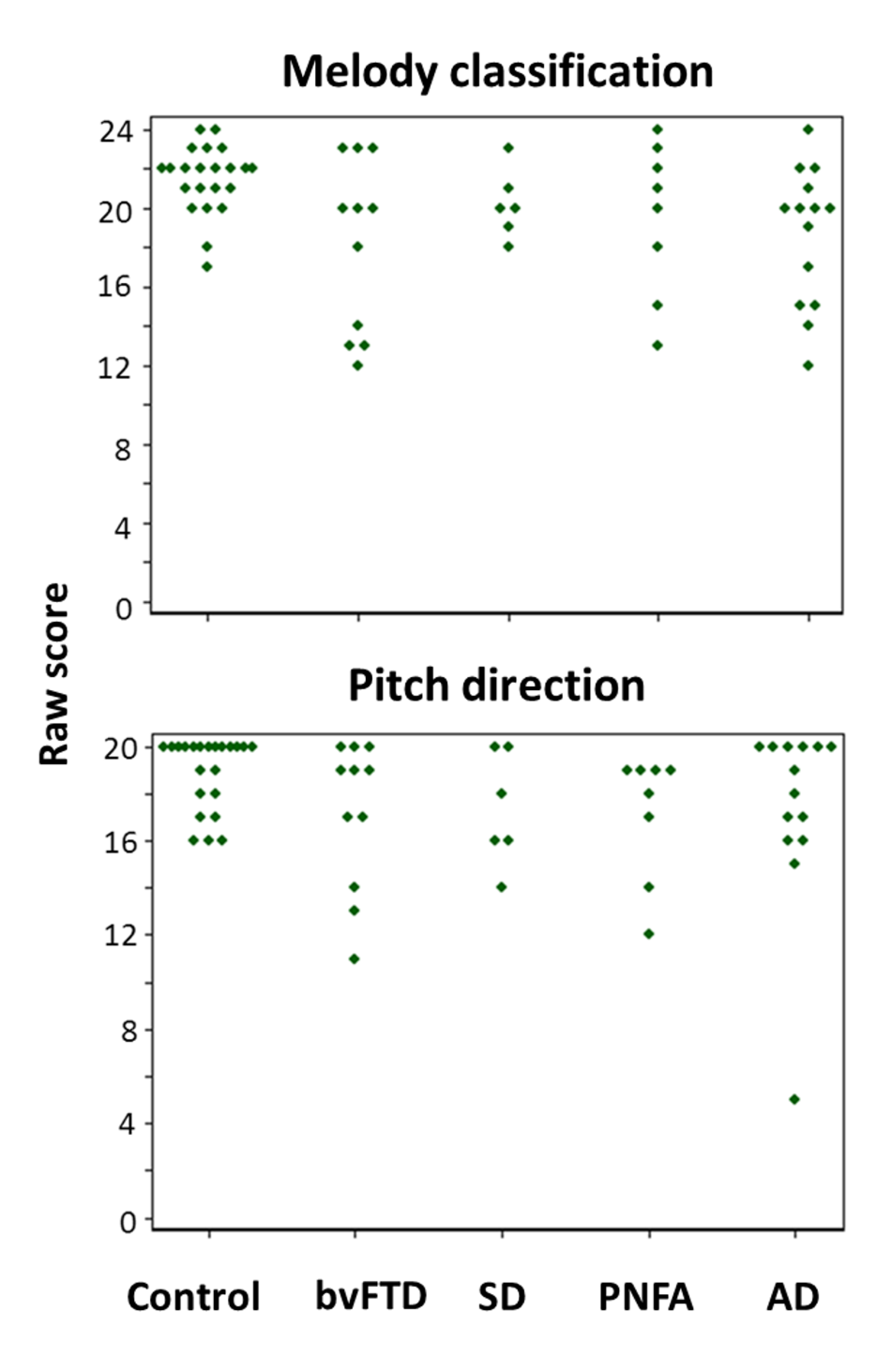


Raw individual accuracy scores for classifying melodies as ‘finished or ‘unfinished’ (above) and determining the direction of pitch changes across sequential tone pairs (below) are shown, for all participants (in both tests, a score of 50% correct signifies chance performance; this corresponds to a score of 12/24 for the melody classification task and 10/20 for the pitch direction task). See text for details of tests. AD, patients with Alzheimer’s disease, bvFTD, patients with behavioural variant frontotemporal dementia, Control, healthy controls; PNFA, patients with progressive nonfluent aphasia; SD, patients with semantic dementia.

**Figure S4.** Individual pleasantness rating scores for all melody stimuli


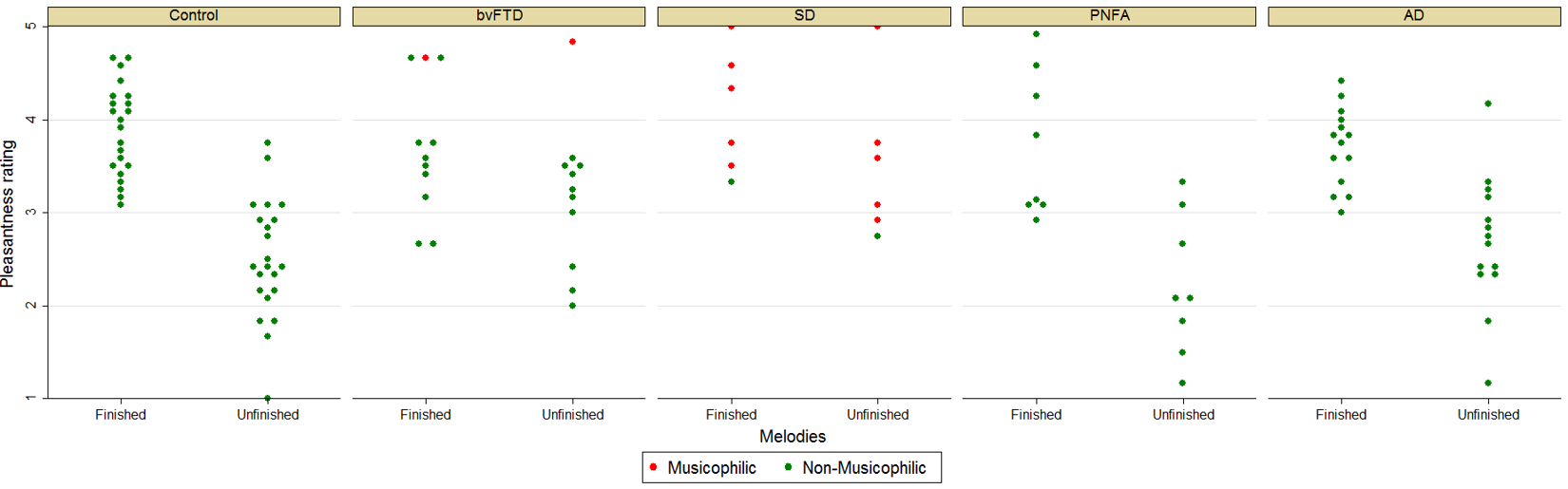


Individual pleasantness rating scores for the complete set of ‘finished’ and ‘unfinished’ melodies are shown for all participants: melody pleasantness was scored on a Likert scale (1, not at all pleasing; 5, very pleasing) and each point represents the mean rating for that participant for that melody ending type. Data for patients with musicophilia are indicated using red dots. AD, patient group with typical Alzheimer’s disease, bvFTD, patient group with behavioural variant frontotemporal dementia, Control, healthy control group; PNFA, patient group with progressive nonfluent aphasia; SD, patient group with semantic dementia.

**Figure S5**. Individual pleasantness rating scores for correctly classified melodies


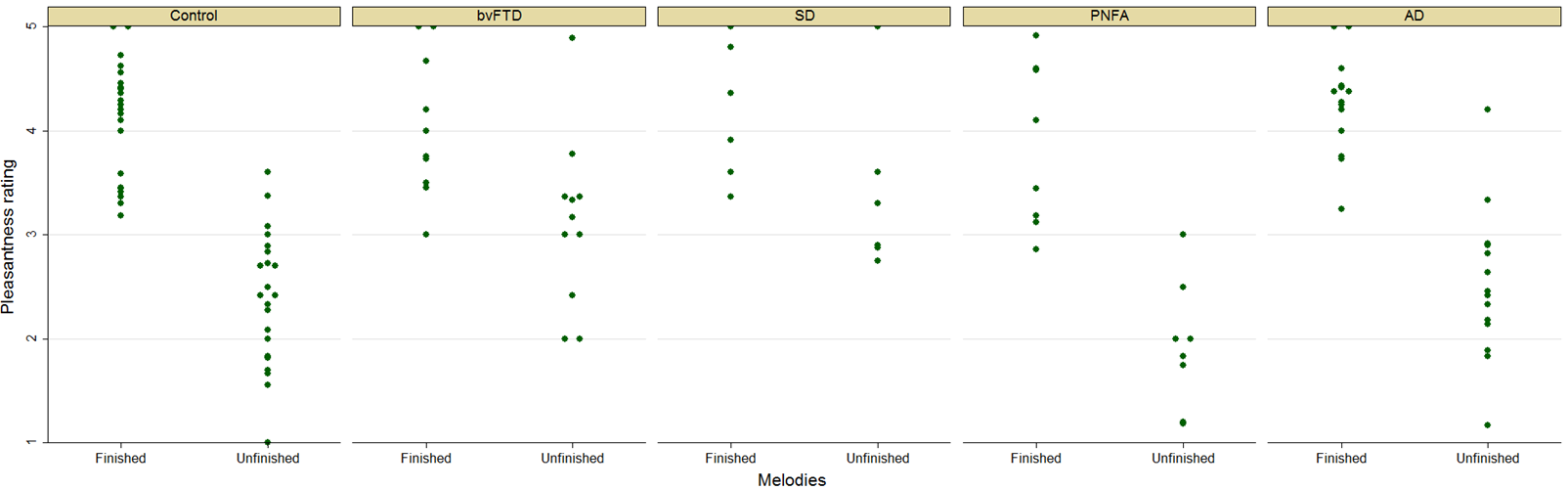


Individual pleasantness ratings of those melodies classified correctly as ‘finished’ or ‘unfinished’ are shown for all participants. Melody pleasantness was scored on a Likert scale (1, not at all pleasing; 5, very pleasing) and each point represents the mean rating for that participant for that melody ending type. The data distributions are similar to those for the complete melody stimulus sets (compare Figure S4). AD, patient group with typical Alzheimer’s disease, bvFTD, patient group with behavioural variant frontotemporal dementia, Control, healthy control group; PNFA, patient group with progressive nonfluent aphasia; SD, patient group with semantic dementia.
